# A feedback loop sustaining neutrophil extracellular trap formation involves S100 proteins, histones, TLR2 and RAGE, and is restrained by albumin

**DOI:** 10.3389/fimmu.2026.1774475

**Published:** 2026-06-01

**Authors:** Vanessa de Carvalho Oliveira, Matthew Mazur, Patrick P. McDonald

**Affiliations:** 1 Pulmonary Division, Medicine Faculty, Université de Sherbrooke, Centre de Recherche du Centre Hospitalier de l’Université de Sherbrooke (CRCHUS), Sherbrooke, QC, Canada; 2 Department of Immunology & Cell Biology, Medicine Faculty, Université de Sherbrooke, and Centre de Recherche du Centre Hospitalier de l’Université de Sherbrooke (CRCHUS), Sherbrooke, QC, Canada; 3 Department of Research, Insmed Incorporated, Bridgewater, NJ, United States

**Keywords:** DAMPs, endogenous factors, histones, human serum albumin, neutrophil extracellular traps, RAGE, S100 proteins, TLR2

## Abstract

Neutrophil extracellular trap (NET) generation must be tightly controlled as this essential antimicrobial response can also cause tissue damage and contribute to various pathologies. We previously described that neutrophils undergoing NET formation release endogenous mediators that feedback on the cells via the RAGE receptor to drive the response. We now identify S100 proteins and histones as prominent endogenous NET inducers. Mass spectrometry analyses of culture supernatant from NETing neutrophils revealed the presence of seven species of S100 proteins and ten histone variants, all of which could bind to RAGE. In addition, endogenous NET inducers were found to act via TLR2 since antagonism of the receptor for up to 3 h post-stimulation hindered NET formation elicited by various classes of physiological stimuli. By comparison, antagonism of TLR4 did not inhibit NET generation. Conversely, we show that serum or equivalent albumin concentrations restrain NET formation by sequestering endogenous NET inducers. Serum depleted of albumin indeed loses its ability to prevent NET formation; likewise, the belated addition of HSA to culture medium blocks NET generation. Prior exposure to serum or albumin renders endogenous NET inducers unable to trigger the response; mass spectrometry analyses confirmed that albumin captures S100 proteins. Collectively, our data unveil a feedback loop of NET formation involving endogenous S100 proteins and histones, acting through RAGE and/or TLR2, that can be physiologically counteracted by albumin.

## Introduction

To sense the presence of pathogens or cellular damage, the immune system relies on pattern-recognition receptors (PRRs), which detect molecules containing pathogen- or damage-associated molecular patterns (PAMPs and DAMPs). Ligation of these receptors activates immune cells, which then release various lipid and protein mediators to orchestrate the immune response. Neutrophils are generally the first immune cells recruited to infection and/or inflammatory sites. Among the various antimicrobial responses that neutrophils can mount, the most drastic is probably the generation of neutrophil extracellular traps (NETs) (1). This process involves the extrusion of decondensed chromatin, alongside cytoplasmic and granular contents, into the extracellular space (2). These extracellular, web-like structures immobilize and inactivate various pathogens (bacteria, viruses, fungi, parasites) (2–4), making NETs a powerful and essential response for containing systemic infections (3, 5).

Beyond their antimicrobial role, the involvement of NETs in various pathological states has become ever more evident. While NETs guard against infections, they may also damage highly vascularized host tissue, such as lungs and kidneys, thus aggravating the deterioration of patients with pulmonary diseases (COVID-19, acute respiratory syndromes, cystic fibrosis, among others) or lupus (6–9). NETs can also promote thrombus formation by serving as a scaffold that activates platelets and coagulation cascades (10, 11). Accordingly, NETs abound in microthrombi, deep vein, and ischemic stroke thrombi (12–14). In the context of cancer, NETs facilitate tumor survival, invasion, and metastasis (15–20). NETs also play a role in autoimmune disorders, by externalizing modified autoantigens involved in systemic lupus erythematosus and rheumatoid arthritis (dsDNA, LL-37, citrullinated proteins, etc.), hence amplifying the pathological cycles of autoimmunity (21, 22). In view of the crucial role of NETs in pathogen control, and of their participation in the pathogenesis of numerous conditions, their release must be tightly regulated.

Various cellular mechanisms driving NET formation have been uncovered. Receptor engagement rapidly mobilizes early-acting kinases that are conserved among various classes of physiological NET inducers, such as TAK1, p38 MAPK and MEK (23, 24). These kinases activate a downstream enzyme that is essential to NET generation: protein arginine deiminase 4/PAD4 (25). In addition, we identified late-acting kinases (Syk, PI3K, PLCγ2) that may be inhibited 2 hours into the process of NET formation and still abrogate the response (23, 26). The latter signaling intermediates appear to mostly control chromatin decondensation and the ensuing dismantlement of the cellular architecture (27–30).

More recent work from our laboratory has revealed an intriguing aspect of NET regulation, i.e. the involvement of endogenous mediators that drive the phenomenon (24). Mass spectrometric analysis of culture supernatants from resting and stimulated neutrophils that had not yet released NETs identified several proteins that were differentially induced, among which was a subset of known or suspected ligands of a common receptor: RAGE. Accordingly, blocking RAGE with an antagonist abrogated NET formation (24). Building upon these findings, the present study delves deeper into the endogenous regulation of NET formation. We now identify S100 proteins and histones as critical endogenous NET inducers. We further reveal that in addition to RAGE, Toll-like receptor 2 (TLR2) also mediates the effect of endogenous NET inducers. Conversely, we also uncover a physiological brake on this process, by demonstrating that human serum albumin (HSA) sequesters endogenous NET inducers, thus preventing them from feeding back on neutrophils.

## Materials and methods

### Antibodies and reagents

Antibodies were used, that were raised against pan-S100 proteins (Abcam ab14849); pan-histones (Sigma #MABE71); calprotectin (R&D Systems #MAB45702); histone H4 (CellSignaling #cs13919); HSP60 (Abcam #ab59457); sRAGE (Viscera Bioscience #A0012-02-100); HSA (Applied Biological Materials #Y107112). An anti-rabbit antibody (AB-105-C), goat serum (AB-108-C), and a mouse IgG1 isotype control (MAB002) were from R&D Systems; mouse IgG2 (5013051) and IgM (14-4752-81) isotype controls were from eBioscience. A TNFα neutralizing antibody was from Peprotech (500-M26). For flow cytometry, antibodes against TLR2-BV605 (ThermoFisher #BD742768), RAGE-Alexa647 (Novus Biologicals #NBP3-11993AF647) and CD66b-PE (Biolegend #305105) were used. Human Fc Block was from BD Pharmingen (#564219). Recombinant human sRAGE was from BioVendor (#RD172116100-HEK) and rh calprotectin from Hycult Biotech (#HC-2021). Various receptor antagonists were also used – LPS-RS for TLR4 (Invivogen #tlrl-prslps), CU-CPT22 for TLR2 (Millipore #614305), and FPS-ZM1 against RAGE (Cayman Chemical #11909). Human serum albumin (fraction V) was from Millipore (#12668). The HSA depletion kit was from Abcam (#ab241023). Among neutrophil stimuli, monosodium urate (MSU) (tlrl-msu) and ultra-pure peptidoglycan (PGN) were from Invivogen (#tlrl-pgnb3); TNFα (#210-TA) and GM-CSF (#7954-GM) were from R&D Systems; and N-formyl-methionyl-phenylalanine (fMLP) was from Millipore Sigma (#F3506). Protein G Sepharose 4FF was from Cytiva (#17-0618-01); Ficoll-Paque Plus from GE Biosciences (Baie d’Urfé, Qc, Canada); Dextran T500 from Pharmacosmos (Holbæk, Denmark); endotoxin-free (< 2 pg/ml) RPMI 1640 from Wisent (St-Bruno, Qc, Canada); poly-L-lysine from Peptides International (Louisville, KY, USA). ProLong Gold antifade reagent, Hoechst 33342, and 16% paraformaldehyde (PFA) were purchased from Thermo Fisher (Missisauga, Canada). PlaNET reagents (fluorescent chromatin-binding polymers) are no longer available from Immune Biosolutions or other suppliers; we therefore employed a close equivalent – fluorescent, 50-nm carboxylate microspheres (#16661-10) from Polysciences Inc. (Warrington, PA, USA). These microspheres are referred to as PlaNET reagents throughout this study, and stain extruded chromatin comparably to anti-histone antibodies (26). All other reagents were of the highest available grade, and all buffers and solutions were prepared using pyrogen-free clinical grade water.

### Cell isolation and culture

Neutrophils were isolated from the peripheral blood of healthy donors, following a protocol that was approved by an institutional ethics review board. All subjects gave written informed consent in accordance with the Declaration of Helsinki. Briefly, whole blood was collected using an anticoagulant (sodium citrate), and successively submitted to dextran sedimentation, Ficoll separation, and water lysis – as described previously (31). The entire procedure was carried out at room temperature and under endotoxin-free conditions. Isolated neutrophils were then resuspended at 5 x 10^6^ cells/ml in RPMI 1640 medium supplemented with 5% autologous serum, at a final concentration of 5 x 10^6^ cells/ml (unless otherwise stated). As determined by Wright staining and FACS analysis, the final neutrophil suspensions contained fewer than 0.1% monocytes or lymphocytes; neutrophil viability exceeded 98% after up to 4 h in culture, as determined by trypan blue exclusion and by Annexin V/propidium iodide FACS analysis. A given blood donor was only used once within a given series of experiments. Thus, the number of independent experiments in a series (as stated in the figure legends) corresponds to the number of different donors.

### NET microscopic assays

Samples were prepared and analyzed exactly as described (26). Unless otherwise stated, we used 2% autologous serum in the culture medium, as in all of our previous studies. The 4-h stimulation time is based on previous studies from our laboratory, and corresponds to when NETs are robustly induced under the conditions that we use (24). For stainings, we previously established that unlike generic DNA dyes (e.g. Sytox Green, DAPI, Hoechst 33342), PlaNET reagents only bind extracellular, decondensed chromatin, thus allowing specific NET staining (23). For quantitation, 3 fields at 10x magnification were counted, that never included the coverslip edges: this amounts to counting about 1,000 neutrophils per coverslip, or about 2000 cells per experimental condition since experiments were conducted in duplicate. Fluorescence quantitation was done using a Java plug-in for ImageJ which we developed (available at http://mcdonaldlab.ca/java-plug-in.html). NET indices were calculated by dividing the NET-specific fluorescence (PlaNET Green) by the number of nuclei (Hoechst 33325) captured in the field of view (10x). The NET indices were then standardized to the positive control of a given experiment.

### Preparation of stimulus-depleted culture supernatants containing endogenous NET inducers

Neutrophils (0.5 mL of 2 x 106 cells/ml in RPMI containing 2% autologous serum) were left to adhere for 1 h on glass coverslips coated with poly-L-lysine. The culture medium was changed to RPMI containing 2% autologous serum and stimulus (either 100 U/ml TNFα or 1 mg/ml monosodium urate crystals (MSU). After 2.5 h of stimulation, the culture medium was collected. Any floating cells were removed by centrifugation (300 g, 10 min, 4 °C). MSU, an insoluble crystal, was precipitated out of solution by centrifugation (18,000 g, 10 min, 4 °C). TNFα was immunodepleted using a neutralizing antibody – 0.1 µg/ml was used to neutralize 100 U/ml (rotating wheel, overnight, 4 °C), the protein G-sepharose beads were added (2 h, room temperature, on a rotating wheel), prior to centrifugation (18,000 g, 10 min, 4 °C). The resulting stimulus-depleted conditioned media (SD-supts) contained only the endogenous mediators released by neutrophils during the stimulation period, as TNFα was no longer detected by mass spectrometry. The SD-supts were aliquoted, snap-frozen in liquid nitrogen, and kept at -20 °C until use.

### Exposure of SD-supts to coated petri dishes

Tissue culture-treated petri dishes (60 x 15 mm, Falcon #25-3002) were coated (or left uncoated) in a culture incubator (37 °C, overnight) with either autologous human serum or with an HSA solution (5 mg/mL in PBS). The next day, excess liquid was removed and the petri dish was washed thrice with PBS so no residual serum or HSA were carried over. SD-supt (1 ml) was added to coated or uncoated petri dishes, which were then placed in culture incubator (2 h, 37 °C). The resulting supernatant was collected and used as NET stimulus.

### Immunoprecipitations

SD-supts were pre-cleared using an antibody (1 µg/mL) from the same species and isotype as the one to be used subsequently to deplete a protein of interest, for 90 min at 4 °C on a rotating wheel. Protein G-sepharose 4FF beads were then added and the SD-supts were incubated another 30 min at 4 °C on a rotating wheel, prior to centrifugation in a microfuge (10 min, 4 °C, maximum speed). The resulting, pre-cleared SD-supts were incubated with 1 µg/ml of antibody raised against a protein of interest (overnight, 4 °C, on a rotating wheel), prior to further incubation with protein G-sepharose beads (60 min, 4 °C, on a rotating wheel) and centrifugation in a microfuge (10 min, 4 °C, maximum speed). The resulting immunoprecipitates were washed once, aliquoted, snap-frozen in liquid nitrogen, and kept at -20 °C until mass spectrometry analysis; whereas the resulting supernatants were aliquoted, snap-frozen in liquid nitrogen, and kept at -20 °C until later use as NET stimulus.

### Immunoblots

Samples were prepared and analyzed exactly as described (26).

### Flow cytometry

Neutrophils (10^6^ pmn/condition) were pelleted (300 g, 10 min, room temperature) and resuspended in 200 µl PBS containing 1% autologous human serum, as well as 1 µg/mL rabbit serum and 1 µg/mL mouse IgG1 isotype control to block any nonspecific binding sites. After a 20-min incubation on ice, the cells were distributed in tubes containing 3 μL of a mouse anti TLR2-BV605 (Becton Dickinson #742768), 12 μL of a rabbit anti RAGE-Alexa647 (Novus Bio #NBP3-11993AF647), or 3 μL of a mouse anti CD66b-PE (Biolegend #305105) and incubated for 30 min on ice. Staining was stopped by adding 800 μL of PBS containing 1% autologous human serum, followed by centrifugation (300 g, 10 min, 4 °C). Cell pellets were resuspended in PBS containing 1% autologous human serum and 0.5 μg/mL propidium iodide. At least 10,000 cells were acquired per experimental condition.

### Mass spectrometry

Pre-cleared immunoprecipitation pellets were prepared in triplicates by using SD-supts from 3 independent donors. On-bead digestion of the proteins and subsequent mass spectrometry analysis were performed by the institutional platform at Université de Sherbrooke, as previously described (32), except for the data in **Table 4** and **Supplementary Table 1**, for which mass spectrometry analyses were performed at Insmed Inc.

### Data analysis

Data are presented as the mean ± s.e.m. of at least three independent experiments; when computing means, any outlying data point (as determined by Grubb’s test) was excluded. When comparing two data groups, statistical significance was assessed using a paired Student’s t-test; for such analyses, data distribution passed the Shapiro-Wilk normality test. For comparisons involving more than two groups, one-way ANOVA was employed, followed by Bonferroni’s *post-hoc* test. All statistical analyses were conducted using Prism 10 software (GraphPad Software, San Diego, CA, USA).

## Results

### Removal of specific protein families from SD-supts abrogates their ability to induce NETs

**Figure 1:**
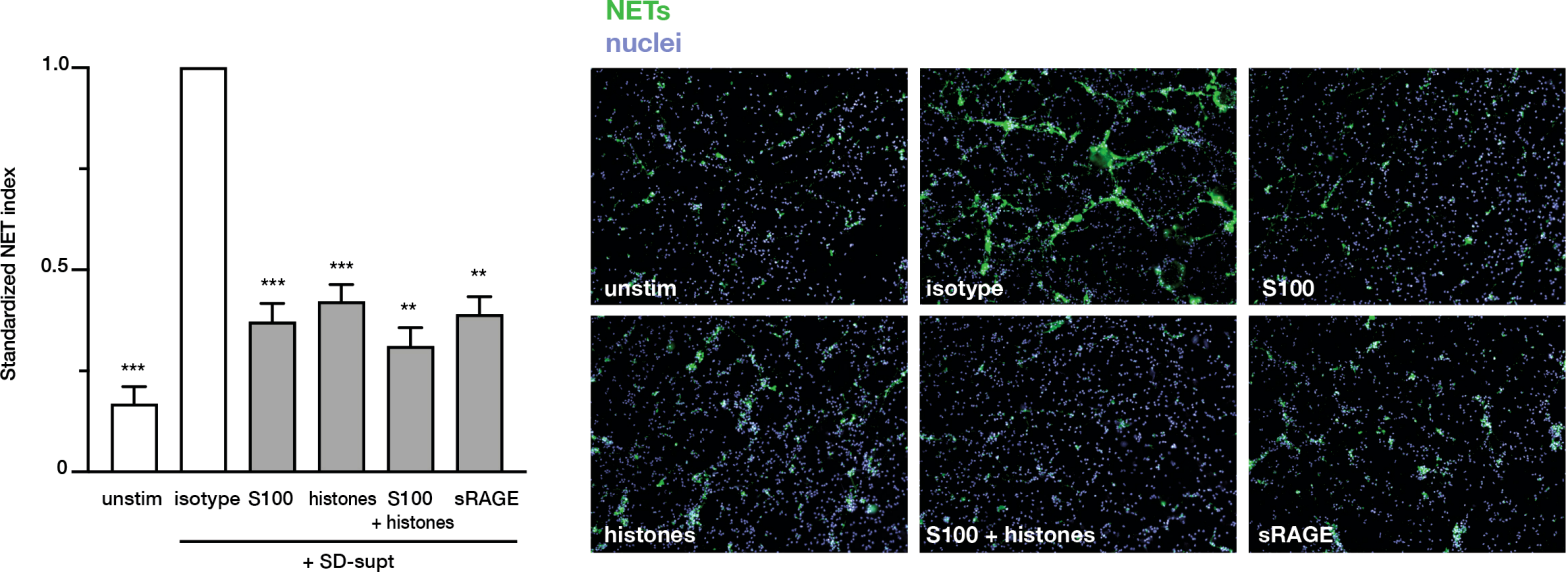
Effect of depleting discrete protein subsets on the ability of SD-supts to induce NETs. Human neutrophils adherent to poly-L-lysine-coated coverslips were stimulated with 1 mg/mL monosodium urate (MSU) for 2.5 h. The culture supernatant was collected and depleted of the original stimulus, yielding a stimulus-depleted culture supernatant (SD-supt). These SD-supts were then pre-cleared and immunoprecipitated using pan-histone (“histones”) or pan-S100 proteins (“S100”) antibodies as described in Methods. Alternatively, SD-supts were pre-cleared, mixed with rh sRAGE, and immunoprecipitated using anti-sRAGE antibodies (“sRAGE”). The resulting supernatants from immunodepleted SD-supts were stored and later used as a NET stimulus. Human neutrophils adherent to poly-L-lysine-coated coverslips were incubated for 4 h at 37 °C in the absence of stimuli (“unstim”) or in the presence of SD-supts that had been only pre-cleared (“isotype”) or immunodepleted of S100 proteins, histones, or sRAGE-bound proteins. NET formation was then assessed by microscopy and standardized NET indices were calculated. A representative experiment is shown (right panel), along with compiled data (mean ± s.e.m.) from at least 3 independent experiments. **, p < 0.01; ***, p< 0.001 vs the positive control; using Student’s paired t test.

We previously demonstrated that endogenous NET inducers are released by neutrophils undergoing NET generation after 2–3 h of stimulation, which feed back on the cells through surface receptors that include RAGE (24). Stimulus-depleted culture supernatants from such NETing neutrophils (thereafter termed SD-supts) were indeed found to contain several potential RAGE ligands (e.g. S100 proteins) and other neutrophil activators (e.g. histones). However, a link between potential NET inducers and an actual participation in driving the phenomenon (though RAGE or other receptors) had not been established. To gain more insight into the issue, SD-supts were immunodepleted of suspected endogenous NET inducers and then tested for their NET-inducing ability. As shown in **Figure 1**, SD-supts that underwent immunoprecipitation with isotype controls retained the capacity to trigger NET formation. By contrast, this property was strongly inhibited when S100 proteins or histones were removed individually or simultaneously. Likewise, pre-incubation of SD-supts with recombinant human soluble RAGE (rh-sRAGE) followed by immunoprecipitation of the sRAGE-ligand complexes, effectively suppressed most of their NET-inducing activity (**Figure 1**). One-way ANOVA statistical analysis revealed that differences among the various immunoprecipitation conditions were not significant (p= 0.3539). Importantly, no one treatment completely prevented NET generation, indicating that the combination of multiple endogenous factors drives the response.

### S100 proteins and histones are essential for NET induction by SD-supts

**Figure 2:**
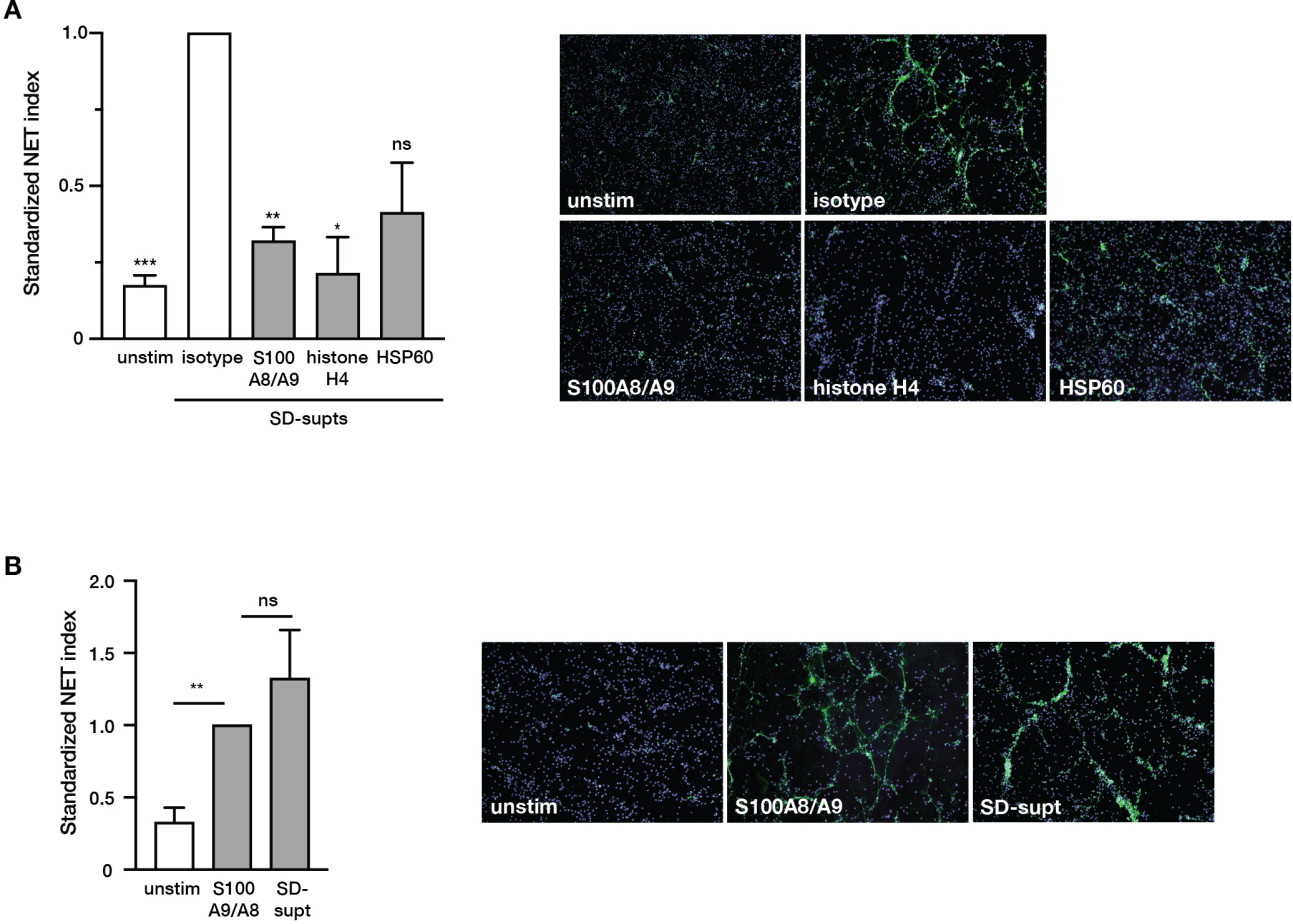
Contribution of individual proteins within SD-supts to their ability to elicit NET formation. **(A)** SD-supts were generated from MSU-stimulated neutrophils, and immunodepleted of individual proteins, as described for **Figure 1**. Human neutrophils adherent to poly-L-lysine-coated coverslips were incubated for 4 h at 37 °C in the absence of stimuli (“unstim”) or in the presence of SD-supts that had been only pre-cleared (“isotype”) or immunodepleted of S100A8/A9, histone H4, or HSP60. NET formation was then assessed by microscopy and standardized NET indices were calculated. A representative experiment is shown (right panel), along with compiled data (mean ± s.e.m.) from at least 3 independent experiments. *, p < 0.05, **, p < 0.01; ***, p< 0.001 vs the positive control; using Student’s paired t test. **(B)** Human neutrophils adherent to poly-L-lysine-coated coverslips were incubated for 4 h at 37 °C in the absence of stimuli (“unstim”) or with 1 μg/mL recombinant human S100A8/A9 dimers or SD-supt from MSU-treated neutrophils. A representative experiment is shown (right panel), along with compiled data (mean ± s.e.m.) from 3 independent experiments. **, p < 0.01; ns, not statistically significant; using Student’s paired t test.

To focus on endogenous proteins that belatedly drive NET production, immunoprecipitates from SD-supts obtained using pan-S100 and pan-histones antibodies were analyzed by mass spectrometry. Seven S100 proteins were thus detected, the most abundant being S100A9, S100A12, and S100A8 (**Table 1**); 10 histones variants were also detected (**Table 2**). When we performed similar analyses of immunoprecipitates from SD-supts obtained using sRAGE, all the above S100 proteins and histones were detected (as listed in **Tables 1**, **2**). Some proteins were exclusively detected in the sRAGE immunoprecipitates, such as the heat-shock protein, HSP-60 (**Table 3**). Some of the proteins identified by mass spectrometry were then individually immunodepleted from SD-supts to determine whether they contribute to the NET-inducing effect of the latter. As shown in **Figure 2A**, removal of calprotectin (S100A8/A9 dimer) and histone H4 from the SD-supts largely eliminated their ability to induce NETs. By comparison, depletion of HSP-60 (a RAGE ligand) from SD-supts did not consistently hinder the NET response (**Figure 2A**). One-way ANOVA statistical analysis revealed that differences among the various immunoprecipitation conditions were not significant (p= 0.5316). When the SD-supts were prepared with a different stimulus (i.e. TNFα instead of MSU), the same endogenous proteins were observed to drive the NET response (**Supplementary Figure 1A**). We confirmed those data by employing a variation of the above experiments, in which NET induction was conducted in the presence of pan-S100 proteins or pan-histones antibodies (or isotype controls) to capture endogenous proteins as they are released. In these experiments, the cells had been pre-treated with FcBlock reagent to prevent NET induction by antigen-antibody complexes via Fc receptors (33). As shown in **Supplementary Figure 1B**, neutralizing endogenous S100 proteins or histones decreased NET formation. Conversely, we tested the ability of an individual endogenous NET inducer to trigger the response in the absence of other SD-supt components. As shown in **Figure 2B**, the S100A8/A9 dimer alone could induce NET formation when added exogenously, although this response tended to be less potent than that achieved by a SD-supt. These data indicate that S100 proteins and histones are the major proteins driving the endogenous feedback loop during NET formation.

**Table 1:** S100 proteins immunoprecipitated from SD-supts.

	pan-S100 proteins + pan-histones			sRAGE		
Protein	expt 1	expt 2	expt 3	expt 1	expt 2	expt 3
Protein S100-A4	1.39E+05	0.00E+00	1.76E+05	5.95E+04	0.00E+00	1.57E+05
Protein S100-A6	5.41E+05	8.31E+04	4.68E+05	6.69E+05	8.13E+04	8.22E+05
Protein S100-A8	2.33E+06	1.25E+06	9.31E+05	3.09E+06	1.20E+06	1.18E+06
Protein S100-A9	9.20E+06	7.70E+06	5.66E+06	1.15E+07	9.20E+06	6.17E+06
Protein S100-A11	4.49E+05	1.28E+05	7.37E+05	6.86E+05	2.30E+05	9.48E+05
Protein S100-A12	2.94E+06	1.32E+06	1.23E+06	4.24E+06	1.96E+06	2.93E+06
Protein S100-P	1.08E+06	4.30E+05	5.81E+05	8.17E+05	4.91E+05	7.73E+05

Pre-cleared SD-supts were immunoprecipitated using a pan-S100 antibody and analyzed by mass spectrometry. Results list the area under the detection peak, a measure of protein abundance.

**Table 2:** Histones immunoprecipitated from SD-supts.

	pan-S100 proteins + pan-histones			sRAGE		
Protein	expt 1	expt 2	expt 3	expt 1	expt 2	expt 3
Histone H1.2	0.00E+00	2.63E+05	0.00E+00	1.12E+05	3.90E+05	0.00E+00
Histone H1.3	5.99E+05	2.76E+05	2.07E+04	5.79E+05	3.55E+05	5.12E+04
Histone H1.4	7.90E+05	7.76E+05	0.00E+00	9.77E+04	0.00E+00	0.00E+00
Histone H1.5	6.59E+05	7.58E+05	1.85E+05	6.31E+05	9.66E+05	2.16E+05
Histone H2A	5.79E+04	1.31E+05	2.19E+05	1.17E+05	2.33E+05	2.02E+05
Histone H2A.V	6.07E+03	3.05E+04	4.16E+04	2.65E+04	6.18E+04	4.07E+04
Histone H2AX	1.91E+05	2.21E+05	3.35E+05	2.83E+05	3.98E+05	4.26E+05
Histone H2B type 1-B	3.10E+04	4.16E+04	5.78E+04	4.90E+04	4.40E+04	9.06E+04
Histone H2B type 1-L	2.49E+05	2.25E+05	2.50E+05	2.85E+05	3.06E+05	4.99E+05
Histone H4	4.93E+04	5.13E+04	8.36E+04	2.23E+05	1.28E+05	3.84E+05

**Table 3:** Proteins from SD-supts exclusively immunoprecipitated by sRAGE.

Protein name	sRAGE expt 1	sRAGE expt 2	sRAGE expt 3
Galectin-3-binding protein	1.89E+04	2.52E+04	1.31E+04
60-kDa heat shock protein, mitochondrial	5.16E+04	3.98E+04	6.55E+04
Heterogeneous nuclear ribonucleoprotein U	4.74E+06	6.30E+06	8.01E+04

Pre-cleared SD-supts were immunoprecipitated using rh sRAGE and an anti-sRAGE antibody, and analyzed by mass spectrometry. Proteins that were consistently detected in all three sRAGE immunoprecipitation experiments, but absent from S100 proteins or histones immunoprecipitates, were considered to be exclusively immunoprecipitated by sRAGE. Results list the area under the detection peak, a measure of protein abundance.

### TLR2 belatedly contributes to the feedback action of endogenous NET inducers, while TLR4 does not

**Figure 3:**
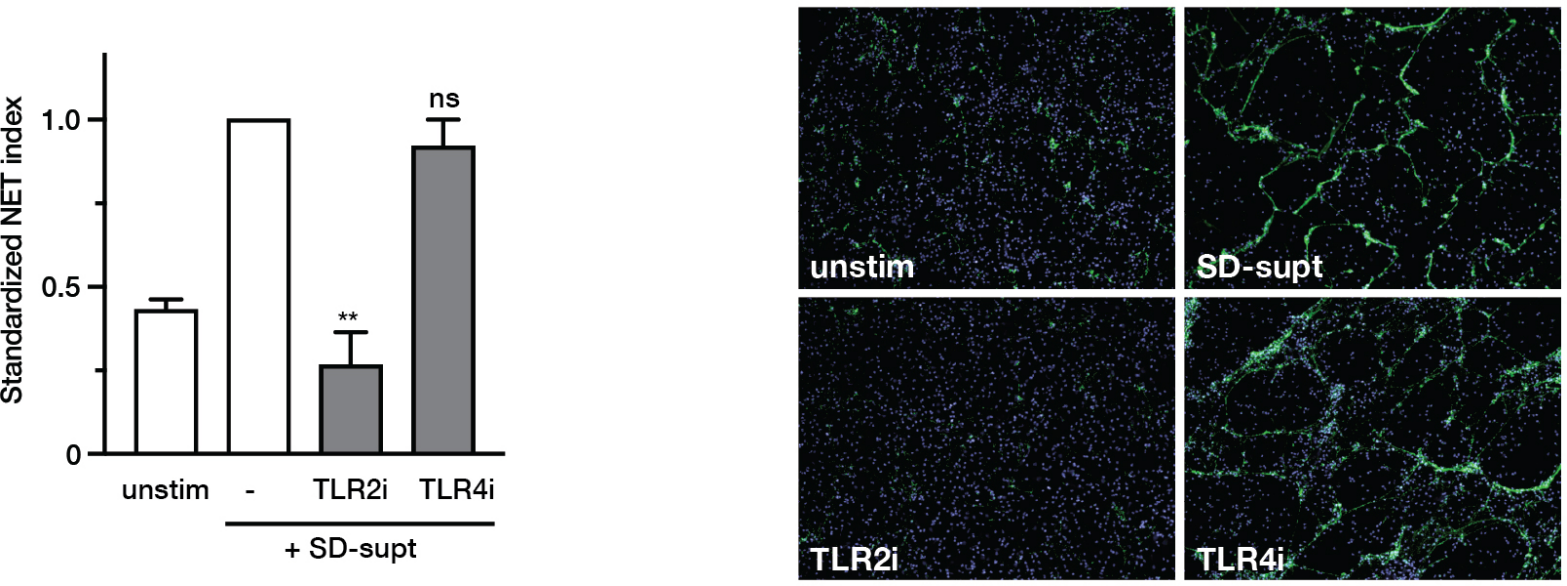
Differential contribution of TLR2 and TLR4 to NET formation driven by endogenous mediators. Human neutrophils cultured on poly-L-lysine-coated coverslips were pre-treated for 15 min with 20 nM CU-CPT22 (a TLR2 antagonist, “TLR2i”), 3 μg/mL LPS-RS (a TLR4 antagonist, “TLR4i”) or their diluents, prior to a 4-h stimulation with SD-supts (prepared from adherent neutrophils exposed to 100 U/ml TNFα for 2.5 h). NET formation was then assessed by microscopy. A representative experiment is shown (right panel), along with compiled data (mean ± s.e.m.) from 3 independent experiments. §, p<0.005 vs unstimulated cells; ns, not significantly different from cells stimulated with SD-supt only; using Student’s paired t test. **, p < 0.01 vs cells stimulated with SD-supt alone.

Because S100 proteins and histones can bind both TLR2 and TLR4 (34–36) in addition to RAGE, we investigated whether these TLRs participate in NET induction by endogenous mediators. To this end, neutrophils were pretreated with a TLR2 antagonist (CU-CPT22) or a TLR4 antagonist (LPS-RS) prior to exposure to SD-supts from TNF-treated neutrophils. As shown in **Figure 3**, blockade of TLR2 prevented NET induction while that of TLR4 had little effect on NET formation elicited by the same SD-supts.

**Figure 4:**
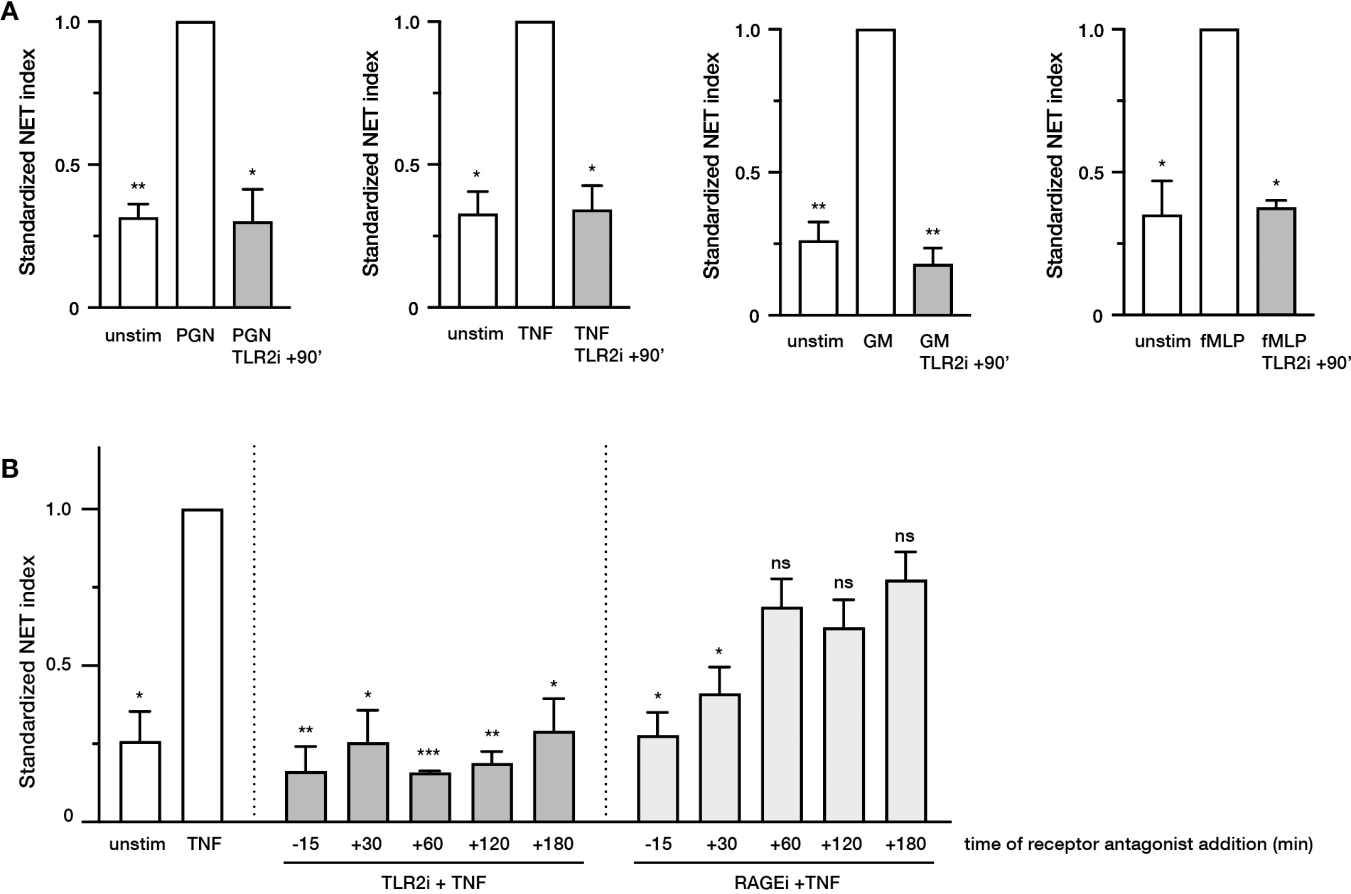
Sequential contribution of RAGE and TLR2 to the belated action of endogenous mediators driving NET formation. **(A)** Human neutrophils adherent to poly-L-lysine-coated coverslips were incubated for 4 h in the absence (“unstim”) or presence of 1 μg/mL ultra-pure peptidoglycan (“PGN”), 100 U/mL TNFα, 1 nM GM-CSF, or 100 nM fMLP. After 90 min of stimulation, a TLR2 receptor antagonist (20 nM CU-CPT22, “TLR2i +90’”) or its diluent was added to the cells. NET formation was then assessed by microscopy. Mean ± s.e.m. of standardized NET indices from 3 independent experiments. **(B)** Human neutrophils adherent to poly-L-lysine-coated coverslips were incubated for 4 h in the absence (“unstim”) or presence of 100 U/mL TNFα. The receptors, TLR2 and RAGE, were antagonized either prior to (-15 min) or after (+30, +60, +120, +180 min) stimulation using 20 nM CU-CPT22 (“TLR2i”) or 1 µM FPS-ZM1 (“RAGEi”). NET formation was then assessed by microscopy. Mean ± s.e.m. of standardized NET indices from 3 independent experiments. *, p < 0.05; **, p < 0.01; ***, p < 0.001 vs the positive control; ns, not significantly different from the positive control; using Student’s paired t test.

Since the requirement for TLR2 was observed using SD-supts, which contain endogenous mediators released late into the process of NET formation (24), we next investigated the effect of adding the TLR2 antagonist belatedly. As shown in **Figure 4A**, adding the TLR2 antagonist 90 min after NET induction largely abrogated NET formation elicited by various classes of exogenous stimuli. Strikingly, antagonizing TLR2 for up to 3 h post-stimulation still effectively blocked NET formation at the 4 h endpoint (**Figure 4B**; **Supplementary Figure 2A**), suggesting that ligation of the receptor by endogenous mediators occurs very late in the endogenous feedback loop. By comparison, adding a RAGE antagonist later than 30 min post-stimulation no longer prevented NET formation (**Figure 4B**), indicating that RAGE ligation by endogenous mediators occurs fairly early in the endogenous feedback loop. One-way ANOVA statistical analysis showed that the timing of addition of the TLR2 antagonist had no significant effect on NET generation (p= 0.4661), while that of RAGE did (p= 0.0083). The same pattern was observed when another exogenous NET stimulus, GM-CSF, was employed (**Supplementary Figure 2B**). Flow cytometry analyses confirmed that all neutrophils isolated from the peripheral blood of healthy individuals express TLR2 and RAGE (**Supplementary Figure 3**).

### Human serum albumin restrains NET formation by sequestering endogenous NET inducers.

**Figure 5:**
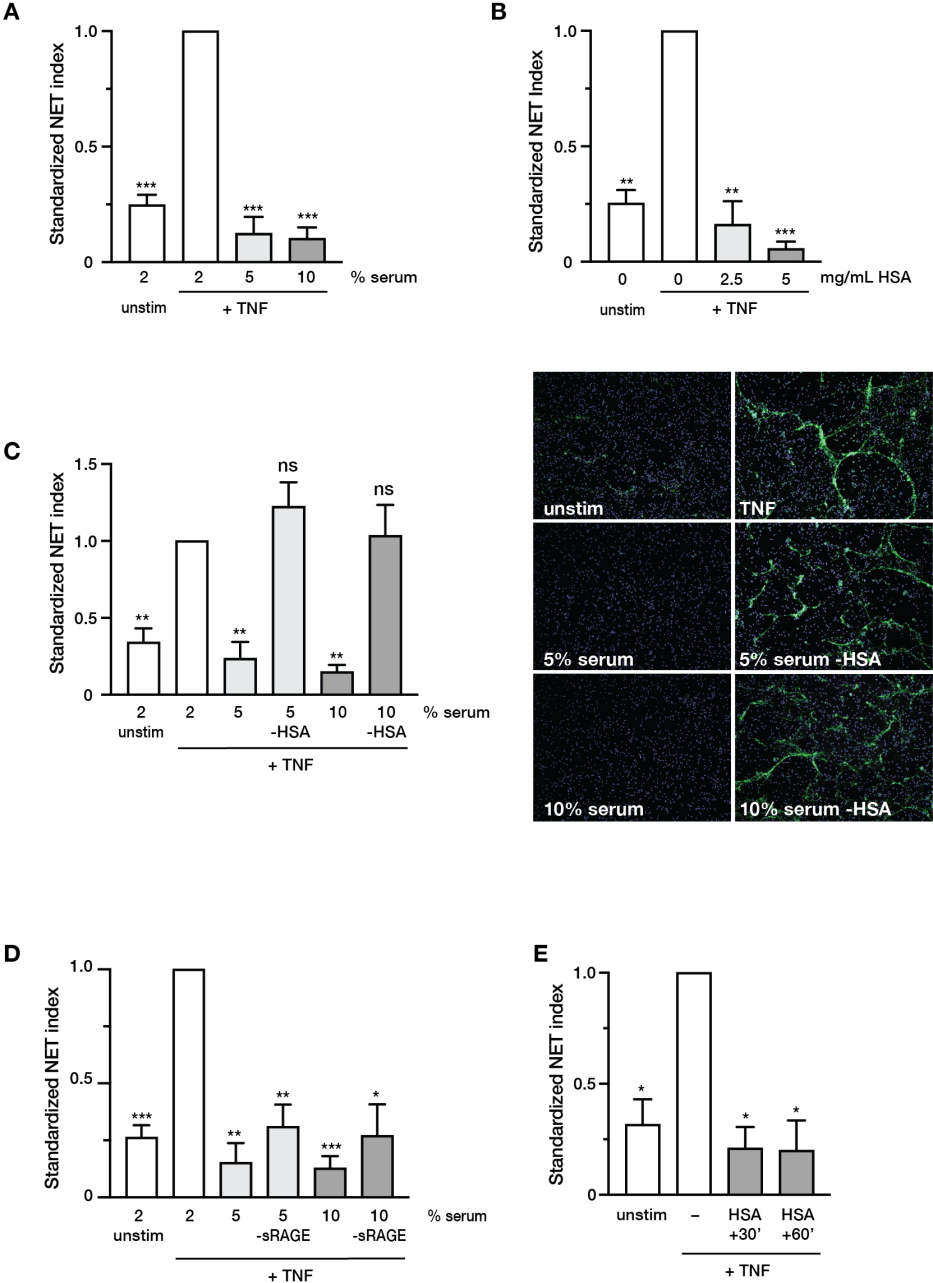
Inhibition of NET formation by serum and HSA. **(A)** Human neutrophils adherent to poly-L-lysine-coated coverslips were incubated for 4 h in the absence (“unstim”) or presence of 100 U/mL TNFα; the culture medium contained the indicated concentrations of autologous serum. NET formation was then assessed by microscopy; mean ± s.e.m. of 3 independent experiments. **(B)** Human neutrophils adherent to poly-L-lysine-coated coverslips were incubated without serum for 4 h in the absence (“unstim”) or presence of 100 U/mL TNFα; the culture medium contained the indicated concentrations of HSA. NET formation was then assessed by microscopy; mean ± s.e.m. of 3 independent experiments. **(C)** Human neutrophils adherent to poly-L-lysine-coated coverslips were incubated for 4 h in the absence (“unstim”) or presence of 100 U/mL TNFα; the culture medium contained the indicated concentrations of autologous serum, which is some cases had been depleted of HSA (“-HSA”). NET formation was then assessed by microscopy. A representative experiment is shown (right panel), along with compiled data (mean ± s.e.m.) from 3 independent experiments (left panel). **(D)** Human neutrophils adherent to poly-L-lysine-coated coverslips were incubated for 4 h in the absence (“unstim”) or presence of 100 U/mL TNFα; the culture medium contained the indicated concentrations of autologous serum, which is some cases had been immunoprecipitated with sRAGE (“-sRAGE”). NET formation was then assessed by microscopy; mean ± s.e.m. of 3 independent experiments. **(E)** Human neutrophils adherent to poly-L-lysine-coated coverslips were incubated without serum for 4 h in the absence (“unstim”) or presence of 100 U/mL TNFα. After 30 or 60 min of stimulation, 50 μL of an HSA solution was added to the cells, yielding a final concentration of 5 mg/mL. NET formation was then assessed by microscopy; mean ± s.e.m. of 3 independent experiments. *, p < 0.05; **, p < 0.01; ***, p < 0.001 vs the positive control; ns, not significantly different from the positive control; using Student’s paired t test.

Because the release of endogenous NET inducers during NET formation acts as an amplification mechanism, it could represent a control point for the phenomenon. In this regard, we and others have reported that beyond a certain threshold, the presence of serum in culture media prevents NET production (23, 37–40). We therefore investigated whether serum components might somehow counteract the effect of endogenous NET inducers. We first validated that the addition of autologous serum to culture media at ≥5% blocks NET release (**Figure 5A**). The most abundant component of human serum is albumin (HSA) and accordingly, the addition of HSA in amounts equivalent to those present in 5 or 10% serum similarly prevented NET generation (**Figure 5B**). To confirm that HSA accounts for most of the NET-inhibiting properties of serum, we depleted HSA from serum using a commercial kit. As shown in **Figure 5C**, the inhibitory effect of serum towards NET generation was completely reverted following HSA depletion. Because human serum also contains sRAGE, which could bind several endogenous NET inducers, we tested whether sRAGE depletion might reverse the inhibitory action of serum on NET formation. As shown in **Figures 5D** however, sRAGE depletion from serum did not rescue NET formation. Finally, we explored the possibility that the presence of HSA might somehow interfere with the initial NET induction, as proposed by Neubert et al. (39). For this purpose, HSA was added to the culture medium either 30 or 60 min after stimulation. As shown in **Figure 5E**, the belated addition of HSA continued to prevent NET formation, in a time frame consistent with the release of endogenous mediators that drive this neutrophil response.

**Figure 6:**
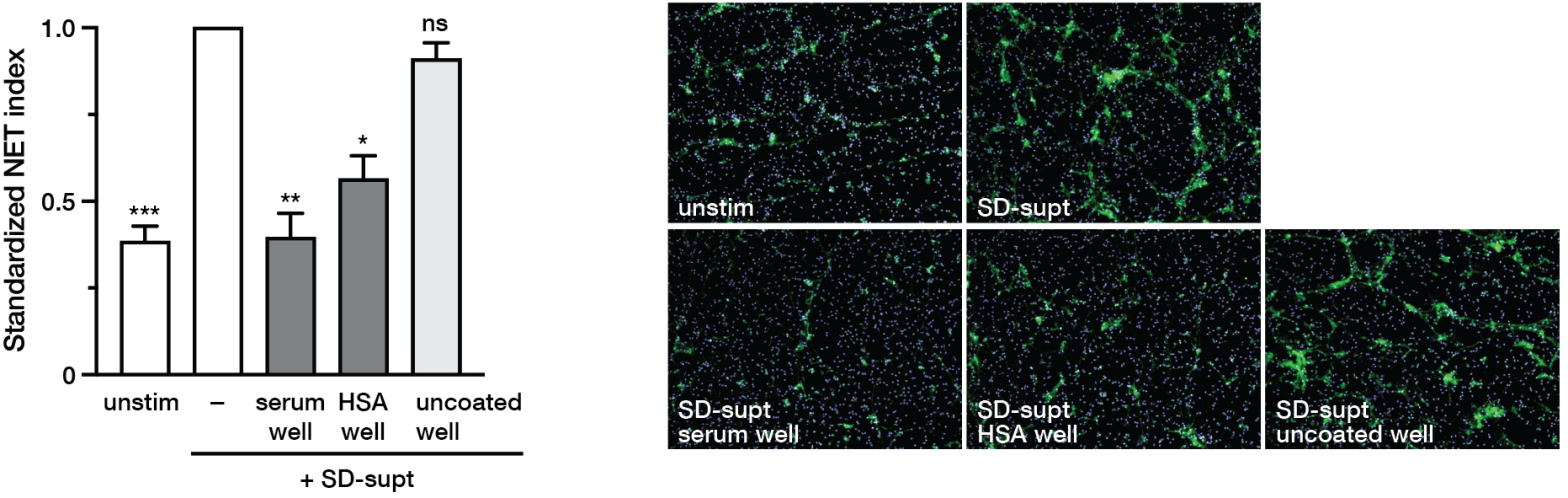
Loss of NET-inducing properties following exposure of SD-supts to serum- or HSA-coated surfaces. Human neutrophils adherent to poly-L-lysine-coated coverslips were incubated for 4 h at 37 °C in the absence of stimuli (“unstim”); in the presence of SD-supts from TNF-treated adherent neutrophils (“SD-supts”); or in the presence of the same SD-supts that had been previously exposed (2 h, 37 °C) to uncoated petri dishes (“uncoated well”) or to petri dishes coated with autologous human serum (“serum well”) or with 5 mg/mL HSA (“HSA well”). NET formation was then assessed by microscopy and standardized NET indices were calculated. A representative experiment is shown (right panel), along with compiled data (mean ± s.e.m.) from 3 independent experiments. *, p < 0.05; **, p < 0.01; ***, p < 0.001 vs the positive control. ns, not significantly different from the positive control.

Given the extraordinary ligand-binding capacity of HSA (41), we tested the hypothesis that HSA inhibits NET production by sequestering endogenous NET inducers. To this end, we coated petri dishes with either human serum or HSA, thoroughly washed off unbound material, and incubated SD-supts in these dishes (2 h, 37 °C). As shown in **Figure 6**, exposure of SD-supts to serum or HSA (5 mg/mL) resulted in a loss of their NET-inducing properties. This was mediated by the coating, as SD-supts incubated in uncoated petri dishes triggered NET production normally. These results suggest that HSA inhibits NET formation by adsorbing endogenous NET inducers. To identify the endogenous mediators captured by HSA, SD-supts from neutrophils stimulated with TNFα or MSU were incubated with HSA (5 mg/mL, 2h, 37 °C) prior to immunoprecipitation using anti-HSA antibodies; the resulting pellets were analyzed by mass spectrometry. As expected, albumin bound many proteins released by neutrophils that are about to extrude NETs (**Supplementary Table 1**). Among these proteins were S100A8, S100A9 and S100A12 (**Table 4**), consistent with the fact that S100 proteins rank among main endogenous drivers of NET formation.

**Table 4:** Myeloid cell-specific proteins in neutrophil SD-supts captured by HSA.

Protein	TNF-treated neutrophils	MSU-treated neutrophils
Protein S100A8	2.70E+08	2.73E+08
Protein S100A9	8.37E+07	6.25E+06
Protein S100A12	1.04E+08	2.62E+07
Galectin-10	7.58E+07	1.17E+08

SD-supts were generated from adherent neutrophils stimulated for 2.5 h with either 100 U/ml TNFα or 1 mg/mL MSU. These SD-supts were pre-cleared with 1 μg/mL rabbit serum and Protein G-sepharose beads, prior to incubation with 5 mg/mL HSA (2 h, 37 °C, under agitation). HSA was then immunoprecipitated using an anti HSA antibody, and the resulting pellets were analyzed by mass spectrometry. Results list the area under the detection peak, a measure of protein abundance. Among the various proteins captured by HSA, only those that are associated with cells of the myeloid lineage are depicted. .

## Discussion

We previously demonstrated that neutrophils undergoing NET formation release endogenous factors that drive this response (24). Analysis of the supernatants from such neutrophils identified S100 proteins and histones (24), which are core NET components (2, 24, 42) and also known to activate various immune cells (43–45). We now show that endogenous S100 proteins and histones are implicated in NET induction since their immunodepletion from culture supernatants of NETing neutrophils abrogated the ability of such supernatants to promote NET formation when added back to fresh neutrophils. Likewise, capturing these proteins as they are being released hindered NET release (**Supplementary Figure 1B**). These findings are consistent with the reported ability of histone H4 and of S100A8/A9 proteins to induce NET generation (24, 43); other members of these protein families may exert a similar effect. Because NET formation was affected to a similar extent when S100 proteins or histones were removed, either individually or collectively, it may be that these proteins act in concert, or perhaps sequentially; there may also be some redundancy among the classes of endogenous mediators that drive NET release. Other proteins released during NET production, such as HSP60, appear to play a lesser role in NET induction. This is in keeping with reports suggesting that HSPs are modulators, rather than strong activators, of immune responses (46–48). High concentrations of S100 proteins and extracellular histones have been detected in several pathologies for which a role for NETs has been extensively reported, including acute injuries, chronic inflammation, autoimmune disorders, and cancer (49, 50). Under these conditions, S100 proteins and extracellular histones may drive NET induction via a feedback loop such as the one described herein. Noteworthy is that these proteins also have antimicrobial properties (2, 4, 51), giving them the potential to act in concert with NETs, in addition to promoting their generation.

We previously established that the receptor, RAGE, is essential to NET induction by various classes of physiological stimuli (24). We now show that RAGE does bind endogenous mediators released during NET formation, that are important for the response. In SD-supts immunoprecipitated using sRAGE (the soluble version of RAGE), which is known to bind the same molecules as the full-length receptor, mass spectrometry analyses of the pellets identified S100 proteins, in addition to HSP60, the glycosylated galectin-3-binding protein, and chromatin components such as HNRNPU (heterogeneous nuclear ribonucleoprotein U) and histones. To our knowledge, this is the first time that these chromatin components are shown to associate with RAGE. This said, RAGE is a pattern recognition receptor that is known to ligate other chromatin components including HMGB1, a non-histone DNA-binding protein (52), and DNA itself (53). Intriguingly, while sRAGE bound histones H1, H2, and H4, it did not bind H3. Other receptors that can bind endogenous NET inducers were found to modulate NET formation. For instance, TLR4 can bind S100A8/A9 and histones (44, 54), but did not participate in NET generation. Conversely, we found TLR2 to be essential for NET induction by endogenous mediators, in a manner that mirrors its ability to elicit NET formation in response to exogenous ligands. We indeed found that adding a TLR2 antagonist up to 3 h after initial cell stimulation blocked NET release. While this is a late time point, the finding remains plausible as the bulk of NET release occurs after the third hour under our experimental conditions (24). By contrast, antagonizing the RAGE receptor beyond 30 min post-stimulation no longer prevented NET release. This indicates that both receptors act at different times post-stimulation, perhaps sequentially, depending on the endogenous mediators being released. Collectively, these results illustrate the complexity with which the NET response can be modulated by endogenous mediators following neutrophil exposure to an initial stimulus.

A novel regulatory mechanism is described herein, whereby HSA adsorbs the endogenous NET inducers, effectively blocking the NET response. An inhibitory effect of serum and HSA on NET formation had been previously reported (23, 37–40). We corroborated those findings and further showed that HSA is primarily responsible for the NET-hindering effect of serum since HSA-depleted serum no longer prevented NET formation; likewise, incubating SD-supts in serum- or HSA-coated plasticware obliterated their NET-inducing properties. Previous studies have proposed that HSA inhibits NET formation by scavenging NET triggers, such as bacterial products (39) or mitochondrial reactive oxygen species (38, 40). We now demonstrate that HSA directly binds S100 proteins, which rank among major endogenous drivers of NET production. The ability of HSA to capture endogenous NET mediators may represent a physiological mechanism to restrain NET formation in the peripheral circulation. Conversely, interstitial fluid and the synovial fluid of inflamed joints, where NETs have been detected, contain much less HSA than plasma (55). As a result, differential HSA concentrations may help ensure that NET formation only occurs under very defined circumstances.

In conclusion, the current work significantly advances our understanding of how NET generation unfolds, by showing that stimulated, adherent neutrophils release S100 proteins and histones, which bind to RAGE and TLR2 receptors to drive NET formation. We also show that this feedback amplification loop can be physiologically neutralized in the presence of serum albumin, which has the ability to sequester S100 proteins. In addition to these phenomena, other mechanisms probably help regulate the NET response. For example, S100A8/A9 can assume an auto-inhibitory conformation that prevents receptor ligation and that is known to restrict local inflammation (56). In view of our findings, a component of this dampened inflammation may well be a deficient NET formation, and this warrants further studies. Another mechanism controlling NET formation may involve the inhibitory MICL receptor, which helps neutrophils recognize extruded DNA, and whose loss or pharmacological inhibition results in uncontrolled NET formation (57). Conversely, when extruded DNA is recognized via the RAGE receptor, IL-8 secretion ensues, which attracts new neutrophils to the site and can directly promote NET generation (23, 58). Clearly, further research is needed to understand how NET regulatory mechanisms interact to fine-tune the response.

## Data Availability

The mass spectrometry data have been deposited to the ProteomeXchange Consortium via the PRIDE partner repository with the dataset identifier PXD078688.
