# Supplementary material for: A feedback loop sustaining neutrophil extracellular trap formation involves S100 proteins, histones, TLR2 and RAGE, and is restrained by albumin
Source: Front Immunol. 2026 Jun 1;17:1774475. doi: 10.3389/fimmu.2026.1774475 (PMC13265349; doi:10.3389/fimmu.2026.1774475)
Supplement: Supplementary file 1 [file DataSheet1.pdf]

**Supplementary Figure 1. Contribution of discrete proteins within SD-supts to their ability to elicit NET formation.** (A) SD-supts were generated from adherent neutrophils stimulated for 2.5 h with either 100 U/ml TNFα, as described for Fig 1. Human neutrophils adherent to poly-L-lysine-coated coverslips were incubated for 4 h at 37°C in the absence of stimuli ("unstim") or in the presence of the aforementioned SD-supts that had been only pre-cleared ("–") or immunodepleted of S100 proteins ("S100"), histones, sRAGE-bound proteins ("sRAGE"), calprotectin ("S100A8/A9"), histone H4, or HSP60. NET formation was then assessed by microscopy and standardized NET indices were calculated. Depicted is data (mean ± s.e.m.) from one experiment performed using duplicate samples. (B) Human neutrophils adherent to poly-L-lysine-coated coverslips were pretreated or not with 2.5 μg Human Fc Block/$10^6$ cells for 15 min ("Fc"), prior to a 4-h stimulation with 100 U/ml TNFα or its diluent ("unstim"), in the presence or absence of 1 μg/mL of pan-S100 protein antibody ("S100"), 1 μg/mL of pan-histone antibody ("histones"), or 1 μg/mL of isotype-matched controls ("isotype"). NET formation was then assessed by microscopy. Depicted is data (mean ± s.e.m.) from one experiment performed using duplicate samples. (C) Control experiment performed to confirm immunoprecipitation specificity. Human neutrophils adherent to poly-L-lysine-coated coverslips were stimulated for 2.5 h with either 100 U/ml TNFα or 1 mg/ml MSU. The resulting SD-supts were incubated with 1 μg/mL of pan-S100 protein antibody ("S100"), 1 μg/mL of pan-histone antibody ("histone"), or 1 μg/mL of isotype-matched control antibody ("iso") overnight at 4°C, under rotation. The samples were subsequently incubated with Protein G-sepharose beads (2h, room temperature, under rotation). Beads-antibody complexes were then precipitated by centrifugation, washed, resuspended in boiling sample buffer, and heated at 95°C for 5 min. For immunoblot analysis, $10^6$ cell-equivalents were loaded per lane. The same pan-S100 and pan-histones antibodies were coupled to biotin using a conjugation kit (Abcam # ab201795) and used as primary antibodies; streptavidin-HRP was employed (Abcam # ab7403) for detection. Whole-cell

neutrophil lysates were used as positive control. Depicted is data from one experiment. xx, empty well (i.e. sample buffer only).

**Supplementary Figure 2. Sequential contribution of RAGE and TLR2 to the NET response.** (A) Human neutrophils adherent to poly-L-lysine-coated coverslips were incubated for 4 h at 37°C in the presence of 1 µg/mL $Pam_3CSK_4$ (a synthetic TLR2 ligand, "Pam"), 100 nM fMLP, or their diluent ("unstim"). After 3 h of stimulation, a TLR2 receptor antagonist (20 nM CU-CPT22, "TLR2i") or its diluent was added to the cells. NET formation was then assessed by microscopy. Depicted is data (mean ± s.e.m.) from one experiment performed using duplicate samples. (B) Human neutrophils adherent to poly-L-lysine-coated coverslips were incubated for 4 h in the absence ("unstim") or presence of 100 nM GM-CSF. The receptors, TLR2 and RAGE, were antagonized either prior to (-15 min) or after (+30, +60, +120, +180 min) stimulation using 20 nM CU-CPT22 ("TLR2i") or 1 µM FPS-ZM1 ("RAGEi"). NET formation was then assessed by microscopy. Depicted is data (mean ± s.e.m.) from one experiment performed using duplicate samples.

**Supplementary Figure 3. Human peripheral blood neutrophils express TLR2 and RAGE.** Human neutrophils were isolated and analyzed for TLR2 and RAGE surface expression by flow cytometry, as described in Methods. (A) The gating strategy sequentially excluded debris and selected for viable, single-cell neutrophils. Given that purified neutrophils were utilized, the cell identity was confirmed via morphological characteristics (FSC vs SSC) – an approach validated by assessing CD66b surface expression, which confirmed a 100% purity within the selected population. Spectral compensation was not required as all samples were stained with a single fluorophore. (B) Positive fluorescence gates were defined using unstained cells to account for baseline autofluorescence. (C) Surface expression of TLR2 and RAGE on human neutrophils, as measured by flow cytometry. Both

the percentage of cells expressing the receptor and the the mean fluorescence intensity (MFI) are indicated. Depicted is data from one experiment.

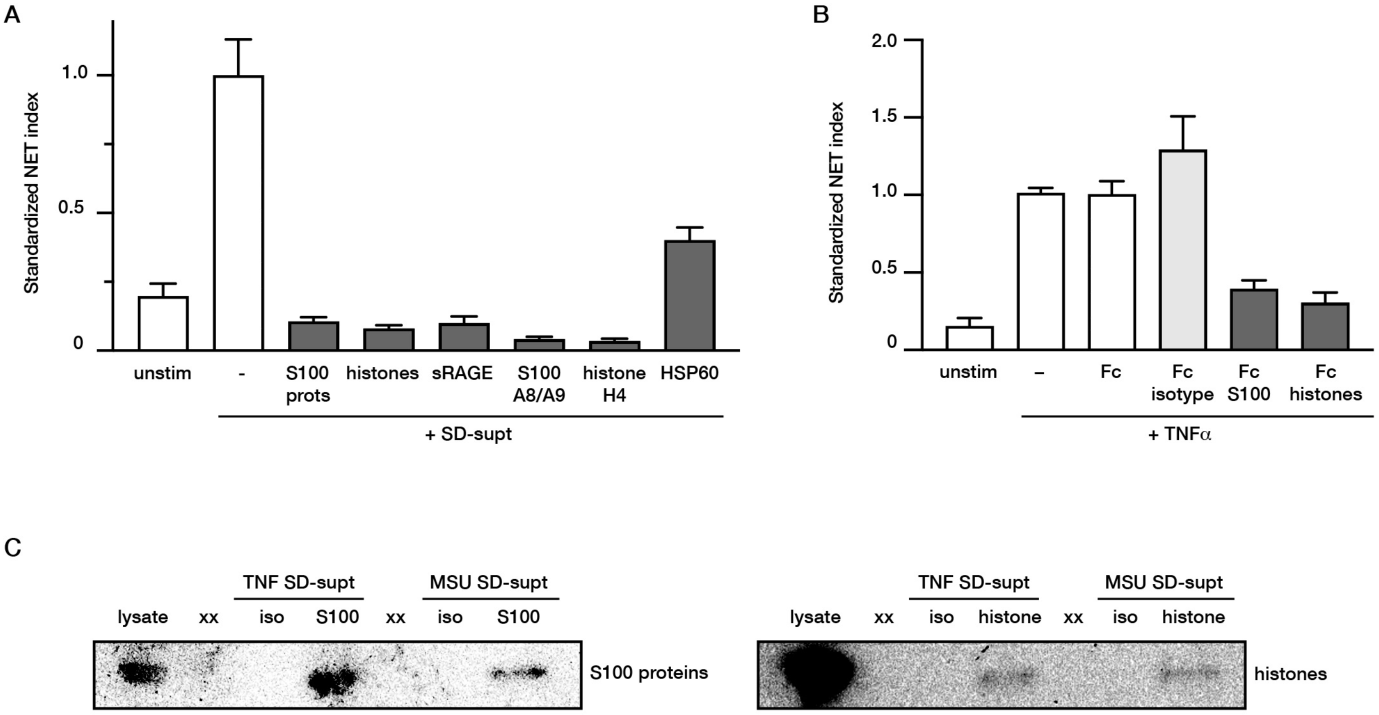


de Carvalho Oliveira *et al.*
Supplementary Fig 1

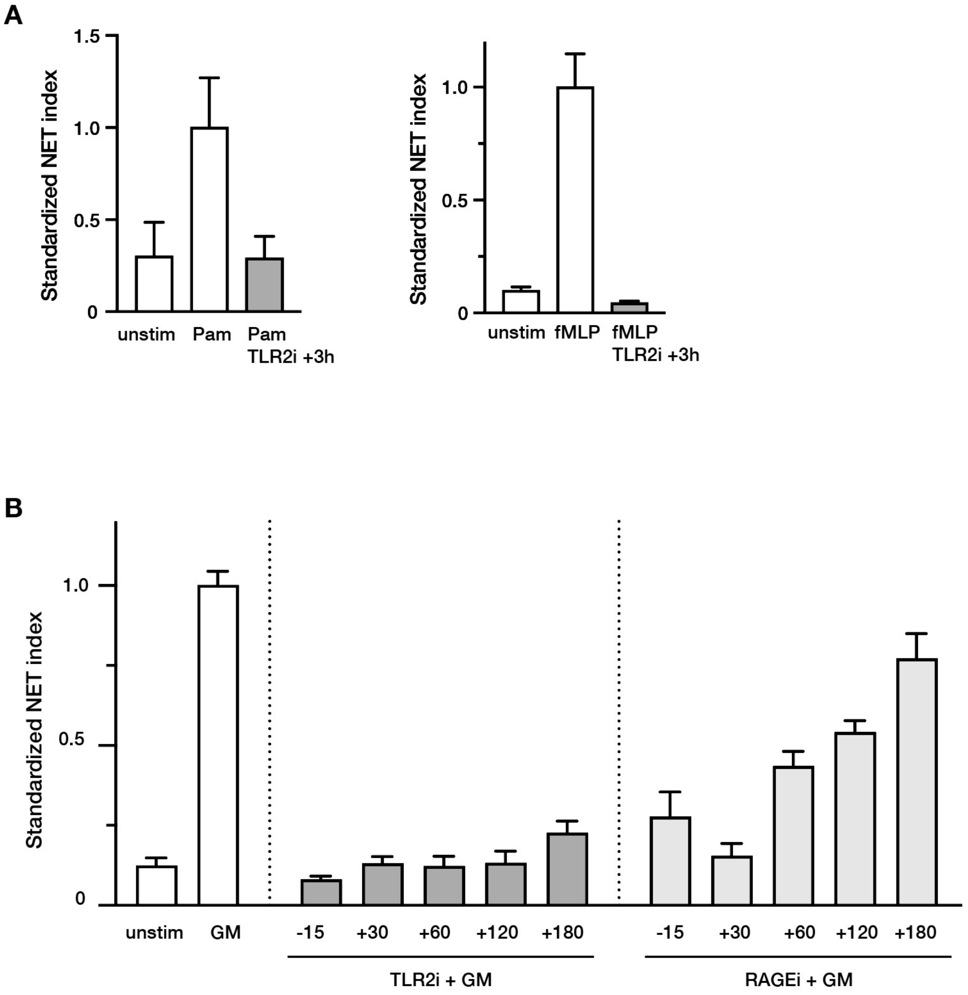


de Carvalho Oliveira *et al.*
Supplementary Fig 2

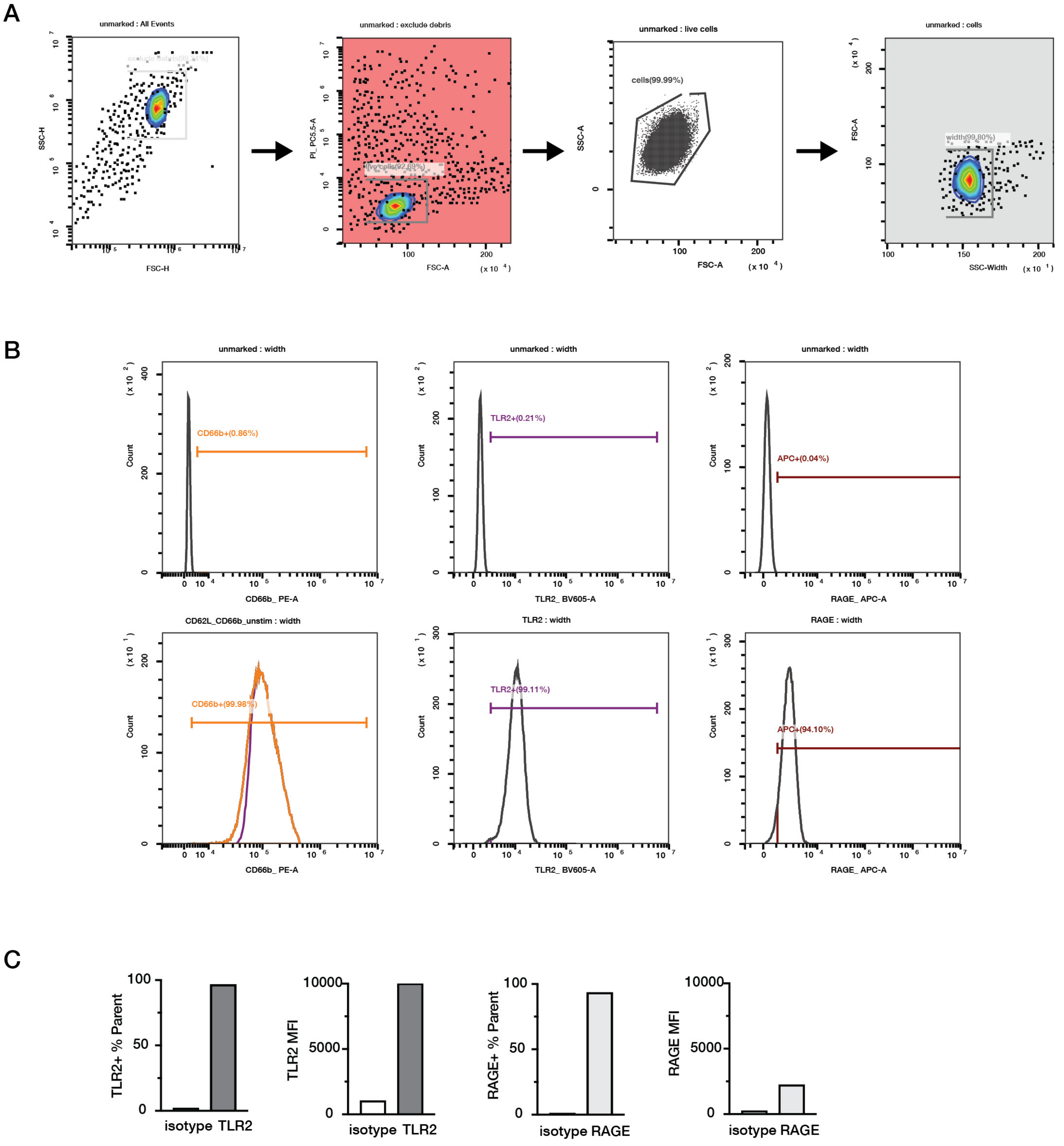


de Carvalho Oliveira *et al.*
Supplementary Fig 3

**Supplementary Table 1. All proteins detected by mass spectrometry analyses of HSA immunoprecipitates from SD-supts.**

SD-supts were generated from adherent neutrophils stimulated for 2.5 h with either 100 U/ml TNFα or 1 mg/mL MSU. These SD-supts were pre-cleared with 1 μg/mL rabbit serum and Protein G-sepharose beads, prior to incubation with 5 mg/mL HSA (2 h, 37°C, under agitation). HSA was then immunoprecipitated using an anti HSA antibody, and the resulting pellets were analyzed by mass spectrometry. Results list the area under the detection peak, a measure of protein abundance.

| Gene name | Protein name | TNF supt | MSU supt | Descriptors |
|---|---|---|---|---|
| ***Myeloid cell-specific proteins*** | | | | |
| S100A12 | Protein S100-A12 | 1.04E+08 | 2.62E+07 | Neutrophil protein |
| S100A8 | Protein S100-A8 | 2.70E+08 | 2.73E+08 | Neutrophil protein |
| S100A9 | Protein S100-A9 | 8.37E+07 | 6.25E+06 | Neutrophil protein |
| CLC | Galectin-10 | 7.58E+07 | 1.17E+08 | Eosinophil protein |
| | | | | |
| ***Plasma proteins*** | | | | |
| AFM | Afamin | 4.42E+09 | 4.23E+09 | Plasma protein |
| ALB | Albumin | 3.42E+12 | 3.28E+12 | Plasma protein |
| HEL-S-153w | Alpha-1-acid glycoprotein 1 | 4.78E+08 | 4.77E+08 | Plasma protein |
| ORM2 | Alpha-1-acid glycoprotein 2 | 4.80E+08 | 9.66E+07 | Plasma protein |
| SERPINA1 | Alpha-1-antitrypsin | 7.20E+07 | 1.76E+07 | Plasma protein |
| A1BG | Alpha-1B-glycoprotein | 4.71E+09 | 4.32E+09 | Plasma protein |

| | | | | |
|---|---|---|---|---|
| AHSG | Alpha-2-HS-glycoprotein | 1.36E+09 | 1.65E+09 | Plasma protein |
| APOA1 | Apolipoprotein A-I | 2.66E+09 | 1.31E+07 | Plasma protein |
| APOA2 | Apolipoprotein A-II | 1.82E+09 | 2.28E+09 | Plasma protein |
| APOC3 | Apolipoprotein C-III | 5.52E+08 | 5.14E+08 | Plasma protein |
| APOD | Apolipoprotein D | 5.97E+07 | 1.56E+07 | Plasma protein |
| APOH | Beta-2-glycoprotein 1 | 5.92E+08 | 7.79E+08 | Plasma protein |
| CP | Ceruloplasmin | 2.83E+08 | 2.32E+08 | Plasma protein |
| C1RL | Complement C1r subcomponent-like protein | 4.42E+07 | 4.00E+07 | Plasma protein |
| C3 | Complement C3 | 2.70E+06 | 3.46E+06 | Plasma protein |
| FETUB | Fetuin-B | 1.17E+07 | 1.37E+07 | Plasma protein |
| HP | Haptoglobin | 4.84E+10 | 4.73E+10 | Plasma protein |
| HPR | Haptoglobin-related protein | 1.58E+09 | 2.55E+07 | Plasma protein |
| HPX | Hemopexin | 1.86E+10 | 1.51E+10 | Plasma protein |
| | Immunoglobulin gamma-1 heavy chain | 1.91E+10 | 1.89E+10 | Plasma protein |
| | Immunoglobulin heavy constant gamma 2 | 7.94E+08 | 5.92E+08 | Plasma protein |
| | Immunoglobulin heavy constant gamma 3 | 3.20E+08 | 2.01E+08 | Plasma protein |
| | Immunoglobulin heavy constant gamma 4 | 3.06E+07 | 7.45E+07 | Plasma protein |

| | | | | |
|---|---|---|---|---|
| IGKC | Immunoglobulin kappa constant | 9.79E+08 | 5.79E+08 | Plasma protein |
| IGLC2 | Immunoglobulin lambda constant 2 | 3.33E+08 | 1.34E+08 | Plasma protein |
| IGLV4-60 | Immunoglobulin lambda variable 4-60 | 1.13E+07 | 9.66E+06 | Plasma protein |
| | Immunoglobulin lambda-1 light chain | 7.50E+06 | 1.29E+08 | Plasma protein |
| KNG1 | Isoform LMW of Kininogen-1 | 8.68E+07 | 1.03E+08 | Plasma protein |
| LTF | Lactotransferrin | 6.88E+07 | 3.74E+07 | Plasma protein |
| LRG1 | Leucine-rich alpha-2-glycoprotein | 4.42E+08 | 3.05E+08 | Plasma protein |
| PGLYRP2 | N-acetylmuramoyl-L-alanine amidase | 4.72E+08 | 4.41E+08 | Plasma protein |
| SERPING1 | Plasma protease C1 inhibitor | 5.24E+07 | 4.49E+07 | Plasma protein |
| AMBP | Protein AMBP | 1.86E+08 | 1.56E+08 | Plasma protein |
| TF | Serotransferrin | 4.50E+08 | 3.55E+08 | Plasma protein |
| SERPINA7 | Thyroxine-binding globulin | 5.11E+07 | 5.90E+07 | Plasma protein |
| SERPINA7 | Transthyretin | 2.92E+09 | 1.03E+09 | Plasma protein |
| GC | Vitamin D-binding protein | 1.02E+08 | 8.11E+07 | Plasma protein |
| AZGP1 | Zinc-alpha-2-glycoprotein | 7.46E+07 | 7.01E+06 | Plasma protein |
| | | | | |

***Other proteins***

| | | | | |
|---|---|---|---|---|
| KRT10 | Keratin, type I cytoskeletal 10 | 1.87E+09 | 1.73E+09 | Cytoskeleton |
| KRT16 | Keratin, type I cytoskeletal 16 | 1.32E+07 | 4.05E+07 | Cytoskeleton |
| KRT9 | Keratin, type I cytoskeletal 9 | 5.01E+08 | 1.44E+09 | Cytoskeleton |
| KRT1 | Keratin, type II cytoskeletal 1 | 1.38E+09 | 3.23E+09 | Cytoskeleton |
| KRT2 | Keratin, type II cytoskeletal 2 epidermal | 8.61E+08 | 1.23E+08 | Cytoskeleton |
| HBA1; HBA2 | Hemoglobin subunit alpha | 2.41E+09 | 1.80E+09 | RBC protein |
| HBB | Hemoglobin subunit beta | 3.74E+09 | 2.86E+09 | RBC protein |
| HBD | Hemoglobin subunit delta | 3.64E+07 | 1.46E+07 | RBC protein |
| SPG11 | Spatacsin | 5.01E+07 | 2.24E+07 | Other intracellular protein |
| SPG11 | Transmembrane protein 198 | 1.74E+09 | 7.91E+08 | Other intracellular protein |
| PRSS1 | Trypsin-1 | 6.52E+07 | 9.32E+08 | Other intracellular protein |
| | Trypsin | 3.19E+10 | 4.25E+10 | Mass spectrometry reagent |
